# The Variation in IRT in Different Ethnic Groups in England—Implications for a Newborn Screening Programme for CF in Diverse Multiethnic Populations

**DOI:** 10.3390/ijns12020028

**Published:** 2026-04-28

**Authors:** Toby Greenfield, Lesley Tetlow, James R. Bonham, Catherine Collingwood, Laura Wainwright, Liz Robinson, Dave Wright, Beverly Hird, Tejswurree Ramgoolam, Caroline Griffith, Lynette Shakespeare, Mehdi Mirzazadeh, Rachelle Garstone, Deborah Finnerty, Nick Flynn, Nazia Taj, Maya Desai

**Affiliations:** 1 Blood Sciences, Portsmouth Hospitals Trust, Portsmouth PO6 3LY, UK; 2 Clinical Biochemistry, Manchester University NHS Foundation Trust, Manchester M13 9WL, UK; lesley.tetlow@mft.nhs.uk (L.T.);; 3 Sheffield Children’s NHS Foundation Trust, Sheffield S10 2TH, UK; 4 Biochemistry Department, Alder Hey Children’s NHS Foundation Trust, Liverpool L12 2AP, UK; 5 NHS England, Wellington House, London SE1 8UG, UK; 6 Institute of Health Research, University of Exeter, Exeter EX4 4PY, UK; 7 Department of Chemical Pathology, Great Ormond Street Hospital for Children, London WC1N 3JH, UK; 8 Specialist Laboratory Medicine, Leeds Teaching Hospitals NHS Trust, St James University Hospital, Leeds LS9 7TF, UK; 9 Department of Chemical Pathology, Epsom & St Helier University Hospitals NHS Trust, Carshalton SM5 1AA, UK; 10 Biochemical Sciences, Synnovis, Guys & St Thomas’ NHSFT, London SE1 7EH, UK; 11 Newborn Screening & Biochemical Genetics Department, Birmingham Women’s and Children’s Hospital NHS Foundation Trust, Birmingham B4 6NH, UK; 12 Biochemical Genetics Unit, Cambridge University Hospitals NHS Foundation Trust, Hills Road, Cambridge CB2 0QQ, UK; 13 Clinical Biochemistry, Oxford University Hospitals NHS Foundation Trust, Oxford OX3 9DU, UK; 14 Department of Respiratory Paediatrics, Birmingham Women’s and Children’s Hospital NHS Foundation Trust, Birmingham B4 6NH, UK; maya.desai@nhs.net

**Keywords:** newborn screening, cystic fibrosis, immuno-reactive trypsin, ethnicity, positive predictive value

## Abstract

Increasing ethnic diversity raises potential inequalities within screening programmes. In the UK, newborns are screened for CF by initially measuring IRT. Dried blood spot IRT levels above a set cut-off require follow-up testing to establish a screening result. Variation exists in IRT levels between different ethnicities and therefore impacts the number of potentially false positive results obtained from ethnic groups. Over a 4-year period, IRT data was collected, and the 99.5th centile was calculated for different ethnic groups. Significant differences were noticed between ethnic groups, and the CF outcome data over a 10-year period were then analysed to establish the effect this had on positive predictive values. The largest difference in IRT 99.5th centile values was seen between the White British and Black African groups. Positive predictive values for Black African and Indian ethnic groups were much lower than the other groups. Rather than try to incorporate ethnicity into the UK CF screening algorithm, we suggest making CF clinicians aware of the differences between different ethnic groups to inform counselling families who receive screen-positive results.

## 1. Introduction

Measurement of immuno-reactive trypsin (IRT) in dried blood spots is the key first step in all newborn screening programmes for cystic fibrosis (CF) in the UK and across Europe [1]. It provides a reliable sensitive biochemical measure of pancreatic dysfunction in the early postnatal period, when blood samples are taken to screen for a number of inherited conditions, including CF. Although CF is a genetic condition, the measurement of IRT as a first step reduces unnecessary genetic tests that may reveal asymptomatic carriers and variants of unknown clinical consequence.

**Figure 1:**
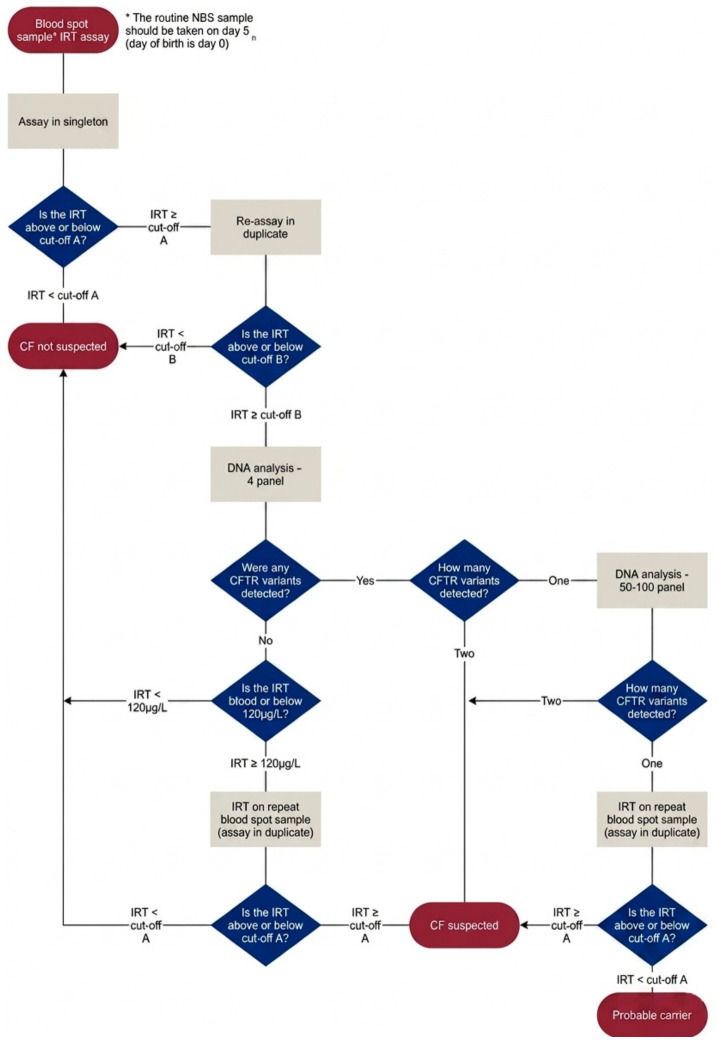
UK cystic fibrosis screening algorithm.

If the result of IRT is above a certain threshold, the sample will be subjected to further testing, including genetics, depending on the specific screening protocol used (see Figure 1 showing the UK CF screening protocol) [2]. In England, the cut-off is taken to be the 99.5th percentile. The results of the subsequent steps in the protocol will determine whether the baby, whose sample it is, is referred to a clinical team with a “CF suspected” result. In certain circumstances, a second IRT cut-off, which is higher than the 99.5th centile, is called upon; the so-called ‘safety net’ arm of the screening protocol. The ability of the initial threshold to reliably distinguish between those babies who are likely to have a CF diagnosis and those babies who do not is key to a well-functioning screening programme.

In 2007, when nationwide screening for CF in England was commenced, the 99.5th percentile was initially set at 70 μg/L, which was calculated from 270,000 samples analysed in five UK screening laboratories [3]. Between 2007 and 2020, cut-offs were continuously adjusted by laboratories that self-monitored their 99.5th percentile. During this period, buddy groups were established where a number of laboratories would be assigned the same reagent lot number. The buddy groups were formed to provide larger data sets that could be utilised by individual laboratories to assist with the accurate assignment of cut-offs. The minimum number of data points for accurate determination of the 99.5th centile has previously been shown to be >10,000 [4]. Due to inherent risks associated with changing cut-offs in complex laboratory software systems and the associated communication requirements, a new approach was introduced in 2020. From 1 April 2020, ‘national’ cut-offs were set based on retrospective data analysis. These cut-offs are reviewed on a quarterly basis by monitoring continued data collection.

We report on the results of the analysis of such data collected between April 2020 and April 2024 with particular reference to IRT values in different ethnic groups. It has been previously reported that IRT levels in newborn babies vary in different ethnic groups [5]. The data collected and analysed show that ethnicity has a significant impact on newborn CF screening outcomes for the population of England.

## 2. Materials and Methods

In England, there are currently two platforms for measuring IRT in bloodspots, both of which use the DELFIA (Dissociation-Enhanced Lanthanide Fluorescence Immunoassay) technology. At the time of writing, the GSP^®^ (Turku, Finland) analyser was being used at Great Ormond Street Hospital (GOSH), SW Thames, Bristol, Cambridge, Sheffield, Manchester and Liverpool (Alder Hey) laboratories. The AutoDelfia^®^ (AD) (Turku, Finland) analyser was being used at South East Thames, Portsmouth, Oxford, Birmingham, Leeds and Newcastle laboratories. Both the GSP and AD are provided by the company Revvity (Waltham, MA, USA)

All 13 laboratories in England, as well as Cardiff and Belfast, submit quarterly IRT data along with the relevant kit Lot numbers used and the number of samples that were referred for DNA testing. Not all laboratories were able to provide ethnicity data due to IT limitations. Ethnicity data was collected from 10 laboratories: GOSH, SW Thames, Cambridge, Sheffield, Manchester, SE Thames, Portsmouth, Oxford, Birmingham and Leeds (see Supplementary Data).

IRT data was collected over a 4-year period from 1 April 2020 to 31 March 2024. Corresponding ethnicity data was also collected. For samples to be included, they had to be taken when babies were 5 to 21 days old and pass National quality checks, including good quality samples, not being taken too close to a blood or platelet transfusion, and not showing signs of contamination, which is indicated by a high variance in results taken from the same bloodspot card. Ethnicity is reported using the Office of National Statistics definition from the 2001 census. 99.5th centile values were calculated for the different groups. Results for the 2 platforms were analysed separately.

For each ethnic group, 99.5th centiles have been plotted graphically, each with 95% confidence interval error bars. For comparison, a cut-off line showing the 99.5th centile for all the data irrespective of ethnicity has been added.

CF outcome data for a 10-year period (April 2014 to end of March 2024) from five English screening laboratories, representing different areas of the country, were merged to assess the impact of ethnicity on the positive predictive value (PPV) and also the CF:CFSPID ratio. Due to the onerous task of retrieving historical outcome data and the time required to process it, only 5 laboratories were asked to contribute.

## 3. Results

### 3.1. IRT Ethnicity Data

The results obtained from the 10 English laboratories are summarised in Table 1 below. The difference between the ethnic groups is very clear, especially between the 99.5th centile seen between White British and Black African. It is noted that the difference between analysers varies, and we can speculate about possible reasons. On the GSP, the Black African group runs 27% higher than the White British, whereas on the AD, the Black African group runs 32% higher than the White British. In addition, the level for the other ethnic groups is spread between that for the White British and Black African babies.

**Figure 2:**
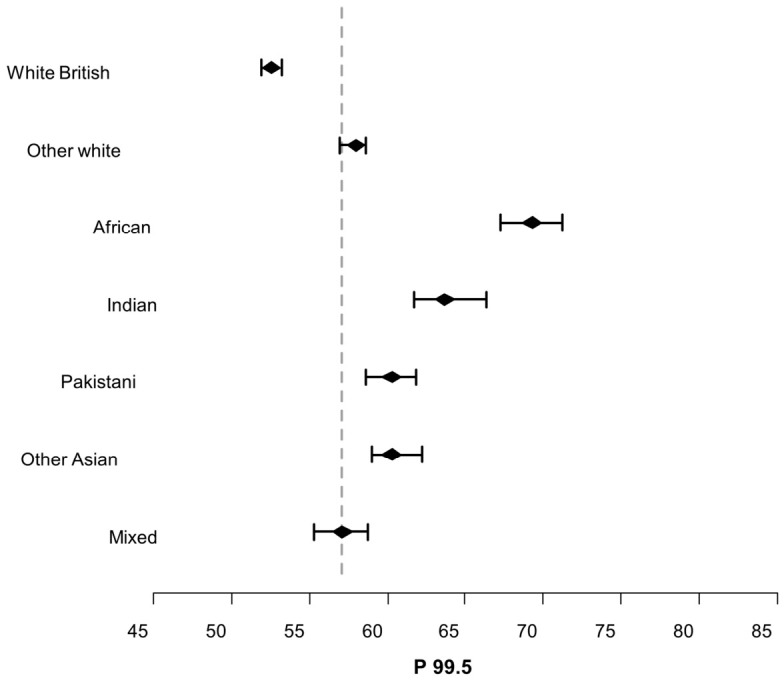
GSP 95% error bars applied to different ethnicities 99.5th centiles.

**Figure 3:**
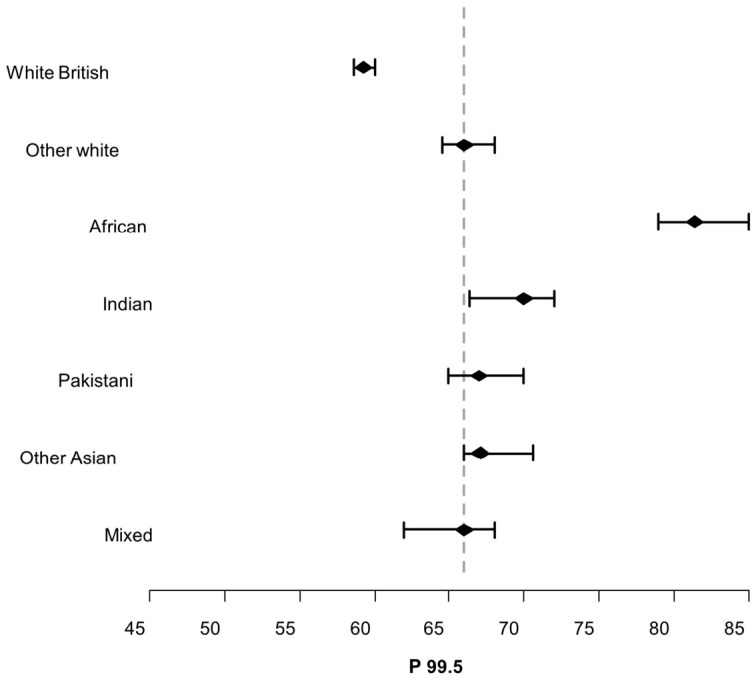
AD 95% error bars applied to different ethnicities 99.5th centiles.

The GSP data is shown in Figure 2 below with 95% error bars applied to each ethnicity’s 99.5th centile (Appendix A Table A1 shows the points in the ordered data where the 99.5th centile and 95% lower and upper confidence intervals were set). A dashed line has been added depicting the population’s 99.5th centile irrespective of ethnicity. Figure 3 is the same but for the AD data (Appendix A Table A2 shows the points in the ordered data where the 99.5th centile and 95% lower and upper confidence intervals were set). The 99.5th error bars shown in Figure 2 and Figure 3 show a similar pattern, with the White British bar being the only group below the population cut-off line. Babies of mixed ethnic backgrounds extend through the cut-off line, while ‘Other White’ and Asian (Indian, Pakistani, and Other Asian) babies’ error bars all appear above the cut-off line. The Black African babies’ 99.5th error bars are appreciably higher than the cut-off line.

### 3.2. CF Outcome Data

The merging of 10 years’ worth of outcome data from five newborn screening laboratories in England (GOSH, Manchester, Birmingham, Sheffield and Portsmouth) is depicted in Table 2 below. It shows that overall, the CF screening programme in England achieves a PPV > 50%. This is above the 30% target that the European Cystic Fibrosis Society (ECFS) has set [6]. The Indian and Black African ethnic groups have PPVs below 20%, which is well below the ECFS 30% target. While we had a total of 1312 screen positives with a known outcome, a further 286 cases with no known outcome have been excluded.

CF Screen Positive, Inconclusive Diagnosis (CFSPID) is the European designation for CF screen positive children who have an inconclusive diagnosis [7]. The general consensus is that newborn screening programmes should aim to minimise the number of CFSPID cases [8]. Across all the ethnic groups, the CF:CFSPID ratio was 7.6:1. The lowest CF:CFSPID ratios were seen in the Indian and Black African ethnic groups, 1.3:1 and 1:1, respectively.

## 4. Discussion

Retrospective analysis of the 99.5th centile for IRT reveals significant ethnic variation, with an increased 99.5th centile being particularly notable in the Black African population, on both the GSP and the AD. For all the 99.5th centile data depicted above, the number of data points exceeded that required for accurate determination of the 99.5th centile and was >300,000 and >26,000 for the White British and Black African groups, respectively.

As both the GSP and the AD are made by Revvity, employing the same technology, you would expect any bias difference in IRT levels measured by these analysers to be constant for all ethnic groups. However, this is not the case. For example, the difference seen for Black African babies between the analysers is 16.1%, compared to 9.4% difference in the Indian babies (Table 1). The reason for the difference is unclear. There are two main IRT isoforms [9]. One possibility is that different IRT isoforms (the same protein but with structural differences) that are being measured may have different prevalence in different ethnic groups, and that there is a difference between the GSP and AD in detecting the two isoforms. Further work on IRT measurement would need to be carried out to try and elucidate this further.

The error bar plots in Figure 2 and Figure 3 show that there is a statistically significant difference between the different ethnic groups, which is most pronounced between the White British and Black African babies (GSP—White British 52.10–53.05, Black African 67.30–71.30, and AD—White British 59.00–60.00, Black African 79.00–85.00). The incidence of CF in the European population, however, is much greater than in the Black African population. A recent study that looked at ancestral diversity of CF within the UK population found that Europeans were five times more likely to have a CF-causing variant than Black Africans [10]. It is apparent that IRT is more likely to be elevated and therefore result in further testing in ethnic groups that are far less likely to go on to have a confirmed CF diagnosis. As alluded to above, it is tempting to postulate that the AD instrument is measuring more of one of the isoforms and that this isoform is more prevalent in Black African babies.

Our study confirms that the ethnic variation in the 99.5th centile increases the number of false positive CF cases in the Black African population. The outcome data in Table 2 shows a markedly higher PPV for White British babies (62.1%) than for the Indian and Black African babies (19.0% and 15.4%, respectively). The lower PPV and therefore higher false positive rate seen in the Indian and Black African babies potentially disadvantages this group via unnecessary further testing. Further still, the CF:CFSPID ratio is the lowest in the Indian and Black African ethnic groups, which reinforces that these groups are at a disadvantage with regard to CF screening. However, due to relatively low numbers, highlighted by the very high ratio seen in the Pakistani group (21:1), we would suggest studies with a larger data set to assess whether the variation in CF:CFSPID ratios in different ethnicities is as significant as this data suggests. It is unfortunate that there were 286 screen positives with an unknown outcome. This highlights the importance of striving to get as near to 100% follow up data for newborn screen positive cases to enable accurate performance monitoring and potential algorithm improvements.

We have considered modifying the UK algorithm to incorporate different IRT cut-offs depending on the ethnicity of the baby. This would however be very difficult to implement reliably in practice as the ethnicity recorded on the bloodspot card is that which is reported by the family [11]. In addition, there may be intermediate cut-off values required in dual heritage cases. Perhaps the most significant obstacle would be maintaining 99.5th centile cut-offs for distinct ethnic groups that ensured statistical validity. We conclude that the most practical solution is to ensure that the professionals who offer counselling to parents who are given a positive CF screen result are knowledgeable about the difference in the risk of CF in children from non-white ethnic groups.

Another option to improve the PPV in the ethnic groups where the gene variants in the current panels are not represented would be to incorporate a larger panel of CF-causing variants or next-generation sequencing (NGS) as a second or third tier test. However, a pilot in the UK of NGS as a second line test designed with this in mind found an increase in the frequency of CFSPID cases, although the study concluded that detection of some of these cases could be avoided by not reporting variants of varying clinical consequence [12]. It would however remain challenging to choose appropriate variants directly relevant to varying ethnic communities which differ around the UK.

With increasing globalisation, it is likely that there will be similar experiences in other countries using IRT to screen for CF, where the diverse population is impacted, for example, in urban areas of France [13].

## 5. Conclusions

Values of IRT vary in babies of different ethnicities, regardless of which DELFIA technology is used (GSP or AD). Babies of Black African heritage have significantly higher IRT values, which we have shown to be associated with a lower PPV. Additionally, babies of Indian heritage also have a low PPV. It is an unusual situation that the analyte we measure to screen for CF is more likely to give screen-positive results in populations in which the disease is much less common. An awareness for clinicians and specialist CF healthcare teams of this ethnic variability, and its impact on the false positive rate in babies who are screen positive, would be beneficial when counselling families.

## Figures and Tables

**Table 1:** IRT 99.5th centiles for ethnic groups on 2 different analysers used for CF screening in 10 newborn screening laboratories in England.

Ethnicity	99.5th Centile GSP	n	99.5th Centile AD	n	% Difference Between GSP & AD
White British	52.6	440,031	58.9	340,546	11.2%
Other White background	58.0	131,006	66.0	62,003	12.9%
African	69.3	40,273	81.4	26,591	16.1%
Indian	63.7	48,525	70.0	22,467	9.4%
Pakistani	60.3	41,259	67.0	35,155	10.5%
Other Asian	60.4	50,936	67.1	22,139	10.5%
Mixed	57.2	36,000	66.0	25,496	14.4%

**Table 2:** Ten years’ worth of CF screening outcome data from five newborn screening laboratories in England.

	White British	Other White	Mixed	Indian	Pakistani	Other Asian	Black African	Any Other	Unknown Ethnicity	Total
Elevated IRT with a known outcome	911	126	65	24	46	33	17	28	62	1312
Total confirmed CF	526	47	29	4	21	11	2	7	31	678
CFSPID	64	9	4	3	1	2	2	2	2	89
CF excluded/carriers	321	70	32	17	24	20	13	12	29	550
CF:CFSPID ratio	8.2	5.2	7.3	1.3	21.0	5.5	1.0	3.5	15.5	7.6
PPV excluding CFSPID	62.1%	40.2%	47.5%	19.0%	46.7%	35.5%	13.3%	26.9%	51.7%	55.4%
PPV including CFSPID	57.7%	37.3%	44.6%	16.7%	45.7%	33.3%	11.8%	25.0%	50.0%	51.7%

## Data Availability

Data will be made available to qualified researchers on request.

## Supplementary Materials

The following supporting information can be downloaded at: https://www.mdpi.com/article/10.3390/ijns12020028/s1.

## Abbreviations

The following abbreviations are used in this manuscript:

CF	Cystic Fibrosis
IRT	Immuno-Reactive Trypsinogen
AD	AutoDelfia
GOSH	Great Ormond Street Hospital
DELFIA	Dissociation-Enhanced Lanthanide Fluorescence Immunoassay
PPV	Positive Predictive Value
CFSPID	Cystic Fibrosis Screen Positive Inconclusive Diagnosis

## Appendix A

**Table A1:** GSP data with where the 99.5th centiles and 95% confidence intervals fall.

GSP Analyser			95% CI	
		P_99.5_	LCL	UCL
Group	n	i	i.lower	i.upper
White British	440,031	437,832	437,739	437,923
Other white	131,006	130,352	130,300	130,402
Black African	40,273	40,073	40,043	40,100
Indian	48,525	48,283	48,251	48,313
Pakistani	41,259	41,054	41,024	41,081
Other Asian	50,936	50,682	50,650	50,713
Mixed	36,000	35,821	35,793	35,847
Total	788,030	784,091	783,967	784,213

**Table A2:** AD data with where the 99.5th centiles and 95% confidence intervals fall.

AD Analyser			95% CI	
		P_99.5_	LCL	UCL
Group	n	i	i.lower	i.upper
White British	340,546	338,844	338,762	338,924
Other white	62,003	61,694	61,658	61,728
Black African	26,591	26,459	26,435	26,481
Indian	22,467	22,356	22,333	22,376
Pakistani	35,155	34,980	34,953	35,006
Other Asian	22,139	22,029	22,007	22,049
Mixed	25,496	25,370	25,346	25,391
Total	534,397	531,726	531,623	531,827
